# Salivary Redox Biomarkers in Insulin Resistance: Preclinical Studies in an Animal Model

**DOI:** 10.1155/2021/3734252

**Published:** 2021-09-09

**Authors:** Mateusz Maciejczyk, Cezary Pawlukianiec, Małgorzata Żendzian-Piotrowska, Jerzy Robert Ładny, Anna Zalewska

**Affiliations:** ^1^Department of Hygiene, Epidemiology and Ergonomics, Medical University of Bialystok, 2C Adama Mickiewicza Street, 15-022 Bialystok, Poland; ^2^Students Scientific Club “Biochemistry of Civilization Diseases” at the Department of Hygiene, Epidemiology and Ergonomics, Medical University of Bialystok, 2c Mickiewicza Street, 15-233 Bialystok, Poland; ^3^Department of Emergency Medicine, Medical University of Bialystok, 2C Adama Mickiewicza Street, 15-022 Bialystok, Poland; ^4^Department of Restorative Dentistry and Experimental Dentistry Laboratory, Medical University of Bialystok, 24A Marii Sklodowskiej-Curie Street, 15-276 Bialystok, Poland

## Abstract

Insulin resistance (IR) is a condition of impaired tissue response to insulin. Although there are many methods to diagnose IR, new biomarkers are still being sought for early and noninvasive diagnosis of the disease. Of particular interest in laboratory diagnostics is saliva collected in a stress-free, noninvasive, and straightforward manner. The purpose of the study was to evaluate the diagnostic utility of salivary redox biomarkers in preclinical studies in an animal model. The study was conducted on 20 male Wistar rats divided into two equal groups: a standard diet and a high-fat diet (HFD). In all rats fed the HFD, IR was confirmed by an elevated homeostasis model assessment (HOMA-IR) index. We have shown that IR is responsible for the depletion of the enzymatic (↓superoxide dismutase) and nonenzymatic (↓ascorbic acid, ↓reduced glutathione (GSH)) antioxidant barrier at both the central (serum/plasma) and salivary gland (saliva) levels. In IR rats, we also demonstrated significantly higher concentrations of protein/lipid oxidation (↑protein carbonyls, ↑4-hydroxynoneal (4-HNE)), glycation (↑advanced glycation end products), and nitration (↑3-nitrotyrosine) products in both saliva and blood plasma. Salivary nonenzymatic antioxidants and oxidative stress products generally correlate with their blood levels, while GSH and 4-HNE have the highest correlation coefficient. Salivary GSH and 4-HNE correlate with body weight and BMI and indices of carbohydrate metabolism (glucose, insulin, HOMA-IR) and proinflammatory adipokines (leptin, resistin, TNF-*α*). These biomarkers differentiate IR from healthy controls with very high sensitivity (100%) and specificity (100%). The high diagnostic utility of salivary GSH and 4-HNE is also confirmed by multivariate regression analysis. Summarizing, saliva can be used to assess the systemic antioxidant status and the intensity of systemic oxidative stress. Salivary GSH and 4-HNE may be potential biomarkers of IR progression. There is a need for human clinical trials to evaluate the diagnostic utility of salivary redox biomarkers in IR conditions.

## 1. Introduction

One of the most significant medical problems of the 21st century is the increased incidence of type 2 diabetes mellitus (DM2). Nowadays, more than 422 million people worldwide have diabetes, of which about 30-40% are still undiagnosed [1]. Several epidemiological studies have shown that DM2 complications cause disability and reduced quality of life for patients with diabetes [1, 2]. DM2 not only leads to micro- and macrovascular angiopathies but is also a significant cause of premature mortality in developed countries [3]. A key role in DM2 development has been attributed to insulin resistance (IR), that is, decreased sensitivity of target tissues to insulin action [4]. IR enhances vascular endothelial proliferation, inflammation, and atherosclerotic plaque formation, mainly due to inhibition of the 3-phosphatidylinositol 3-kinase (PI-3K) and mitogen-activated protein kinase (MAP-kinases) pathways. In addition, IR is inextricably linked to obesity [4, 5]. Increased levels of free fatty acids (FFAs), adipokines, and proinflammatory cytokines inhibit insulin action by affecting the insulin receptor substrate (IRS-1) or impairing translocation of the glucose transporter GLUT4 [4–6]. Nevertheless, the common denominator of metabolic disorders in obesity, IR, and DM2 is also oxidative stress. Increased formation of reactive oxygen species (ROS) occurs not only under ceramide, diacylglycerol (DAG), and triacylglycerol (TAG) accumulation but also impaired insulin signaling and inflammation [7–9]. Oxidative stress has been shown to increase the expression of stress-activated kinases such as protein kinase C (PKC) and c-Jun N-terminal kinase (JNK), which blocks phosphorylation of IRS-1 tyrosine residues and thus leads to increased blood glucose levels [6, 9, 10]. Overproduction of ROS also results in the phosphorylation of NF-*κ*B (nuclear factor kappa-light-chain-enhancer of activated B cells) inhibitor, responsible for activating this transcription factor and inducing inflammation [6, 11]. It also enhances the synthesis of ceramide and, through positive feedback, increases the production of free radicals [12–14]. Therefore, oxidative stress is considered one of the most important pathological factors in the progression of metabolic diseases [6, 9, 10, 15, 16]. Therefore, it is not surprising that redox biomarkers have been postulated for the diagnosis of IR [17–22].

A biomarker is an objectively measurable change in a biological material, which may indicate a normal physiological state (clinical diagnosis), a pathological state (monitoring of disease progression), or a response to pharmacological treatment (evaluating the effectiveness of drug therapy) [23]. However, despite the tremendous development of diabetology and endocrinology, the current state of knowledge is still not sufficient to rapidly diagnose and treat the metabolic complications of IR. Therefore, alternative and effective diagnostic/treatment methods are constantly being sought [1]. Recently, saliva is of increasing interest in clinical diagnostics. Its advantages include low cost, high durability, and noninvasive and painless collection, including from children and disabled persons [24–27]. Although the main component of saliva is water, it also contains electrolytes, amino acids, proteins, lipids, hormones, vitamins, antioxidants, and oxidation products of biomolecules [28]. These compounds can pass from the blood to saliva by passive (simple diffusion, ultrafiltration, and facilitated diffusion) and active transport as well as through damaged cell membranes [29, 30]. Thus, saliva contains most of the compounds present in the plasma, which is the basis for its use in medical diagnostics. Despite many studies on the role of oxidative stress in the pathogenesis of IR [6, 9, 10, 15, 16], there are no data on the usefulness of salivary redox biomarkers in the diagnosis of insulin resistance. Therefore, our study is the first to evaluate the clinical utility of salivary antioxidants and oxidative stress products in a preclinical study in an animal model. In insulin-resistant rats, the activity/concentration of salivary redox biomarkers was compared with their plasma and serum levels and the classical biomarkers of metabolic disturbances.

## 2. Results

### 2.1. Animal Characteristics

The study was conducted on 20 male Wistar rats divided into two equal groups: a standard diet and a high-fat diet (HFD). Body weight, BMI, and energy intake were significantly higher in the HFD group than in the control group. Interestingly, the food intake in rats fed HFD was notably lower compared to the normally fed rats. Moreover, plasma glucose, insulin, leptin, and resistin concentrations were significantly higher in the HFD group compared to the control. HOMA-IR index was significantly higher in all HFD-fed animals confirming systemic IR (Table 1).

### 2.2. Enzymatic Antioxidants

Antioxidants are compounds that form a protective barrier in cells, neutralizing the effects of free radicals. Antioxidant enzymes include catalase (CAT), salivary peroxidase (Px)/glutathione peroxidase (GPx), glutathione reductase (GR), and superoxide dismutase (SOD).

**Figure 1:**
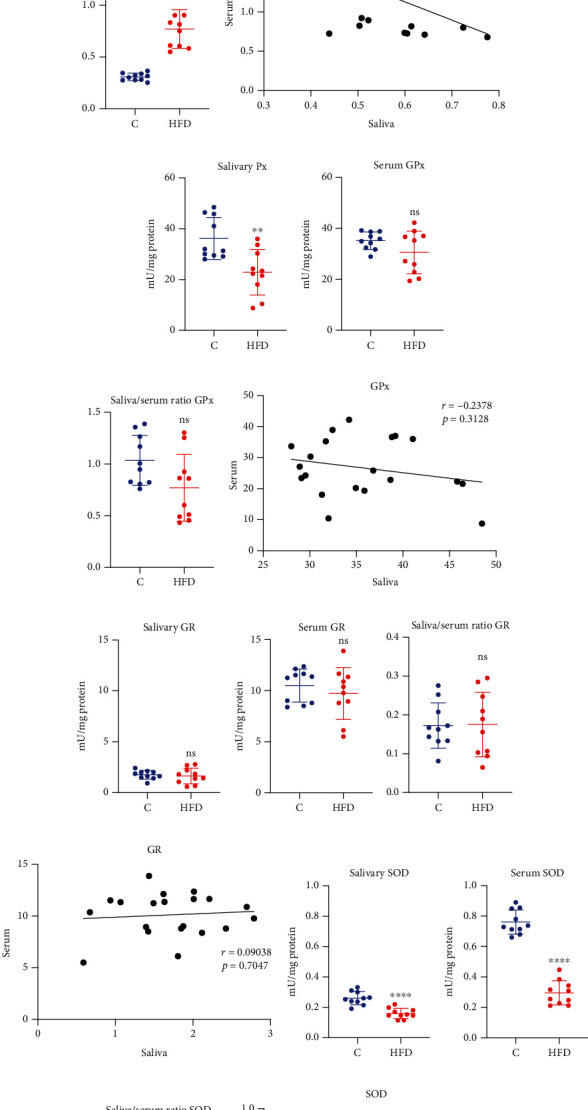
The effect of a high-fat diet (HFD) on salivary and plasma enzymatic antioxidants in rats. Abbreviations: C: control group; HFD: high-fat-diet group; CAT: catalase; GPx: glutathione peroxidase; GR: glutathione reductase; Px: peroxidase; SOD: superoxide dismutase; ^∗∗^*p* < 0.01 vs. control; ^∗∗∗∗^*p* < 0.0001 vs. control; ns: no significance.

The analysis of enzymatic antioxidants revealed a statistically significant decrease in the activity of salivary Px (↓36%, *p* = 0.0029, Figure 1(e)) and salivary SOD (↓39%, *p* ≤ 0.0001, Figure 1(m)), as well as serum CAT (↓53%, *p* ≤ 0.0001, Figure 1(b)) and serum SOD (↓61%, *p* ≤ 0.0001, Figure 1(n)) in the HFD group compared to the control. On the other hand, no notable difference in the activity of salivary CAT (Figure 1(a)), salivary GR (Figure 1(i)), serum GPx (Figure 1(f)), and serum GR (Figure 1(j)) between groups was observed.

Moreover, significantly higher saliva/serum ratio of CAT (↑150%, *p* ≤ 0.0001, Figure 1(c)) and SOD (64%, *p* ≤ 0.0012, Figure 1(o)) in HFD rats was observed in comparison to the control group.

What is more, we observed a high positive correlation of SOD activity (*r* = 0.7501, *p* = 0.0001, Figure 1(p)) between serum and saliva in HFD rats. There were no notable correlations of the other investigated enzymatic biomarkers.

### 2.3. Nonenzymatic Antioxidant

**Figure 2:**
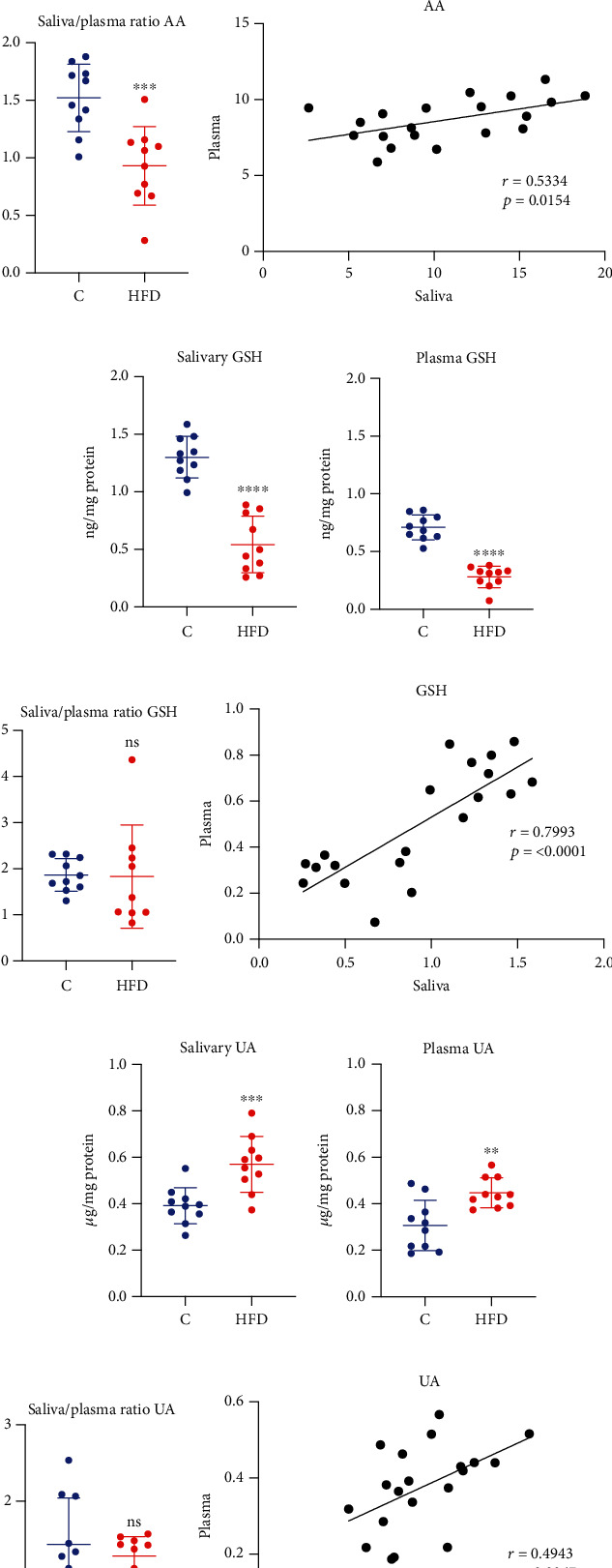
The effect of a high-fat diet (HFD) on salivary and plasma nonenzymatic antioxidants in rats. Abbreviations: C: control group; HFD: high-fat-diet group; AA: ascorbic acid; GSH: reduced glutathione; UA: uric acid; ^∗^*p* < 0.05 vs. control; ^∗∗^*p* < 0.01 vs. control; ^∗∗∗^*p* < 0.001 vs. control; ^∗∗∗∗^*p* < 0.0001 vs. control; ns: no significance.

Nonenzymatic antioxidants mainly include small molecular weight antioxidants such as ascorbic acid (AA), reduced glutathione (GSH), and uric acid (UA). Assays evaluating nonenzymatic antioxidant revealed that high-fat diet caused a significant decrease in the level of salivary (↓52%, *p* ≤ 0.0001, Figure 2(a)) and plasma AA (↓19%, *p* = 0.0014, Figure 2(b)). Similarly, GSH concentration in the saliva (↓58%, *p* ≤ 0.0001, Figure 2(e)) and in the plasma (↓61%, *p* ≤ 0.0001, Figure 2(f)) in the HFD group was significantly lower than in the control group. What is more, a high-fat diet increased the concentration of UA in the saliva (↑46%, *p* = 0.001, Figure 2(i)) and the plasma (↑46%, *p* = 0.0024, Figure 2(j)) of HFD rats comparing to the control.

A significantly lower saliva/serum ratio of AA (↓39%, *p* = 0.0006, Figure 2(c)) in rats fed a high-fat diet was observed compared to the control group. Interestingly, there were no statistical differences in the saliva/plasma ratio of GSH (Figure 2(g)) and UA (Figure 2(k)).

Moreover, a highly positive correlation of GSH content (*r* = 0.7993, *p* ≤ 0.0001, Figure 2(h)) in plasma and saliva of HFD rats was noticed. AA (*r* = 0.5334, *p* = 0.0154, Figure 2(d)) and UA (*r* = 0.4943, *p* = 0.0267, Figure 2(l)) levels were also positively correlated between rats' plasma and saliva.

### 2.4. Oxidative/Nitrosative Damage

**Figure 3:**
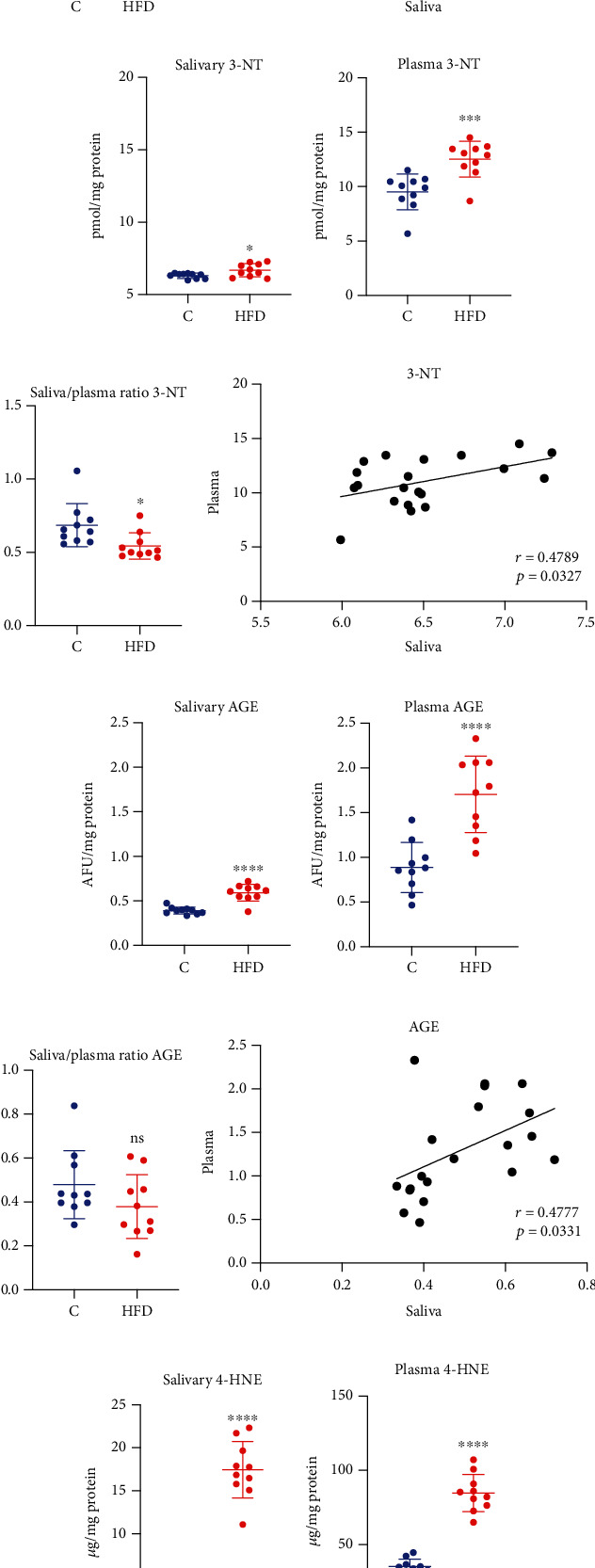
The effect of a high-fat diet (HFD) on salivary and plasma oxidative/nitrosative damage in rats. Abbreviations: C: control group; HFD: high-fat-diet group; 3-NT: 3-nitrotyrosine; 4-HNE: 4-hydroxynonenal; AGE: advanced glycation end products; PC: protein carbonyls; ^∗^*p* < 0.05 vs. control; ^∗∗^*p* < 0.01 vs. control; ^∗∗∗^*p* < 0.001 vs. control; ^∗∗∗∗^*p* < 0.0001 vs. control; ns: no significance.

Oxidation (carbonyl groups (PC); 4-hydroxynoneal (4-HNE)), glycation (advanced glycation end products (AGE) and nitration (3-nitrotyrosine (3-NT)) products of proteins and lipids are used to assess the damage caused by ROS and RNS. The oxidative and nitrosative stress markers revealed that high-fat diet increased concentrations of salivary, as well as plasma PC (↑71%, *p* = 0.0018, Figure 3(a); ↑46%, *p* ≤ 0.0001, Figure 3(b), respectively), 3-NT (↑6%, *p* = 0.0235, Figure 3(e); ↑31%, *p* = 0.0007, Figure 3(f)), AGE (↑51% *p* ≤ 0.0001, Figure 3(i); ↑92%, *p* ≤ 0.0001, Figure 3(j)), and 4-HNE (↑292% *p* ≤ 0.0001, Figure 3(m); ↑140%, *p* ≤ 0.0001, Figure 3(n)).

A significantly lower saliva/serum ratio of 3-NT (↓21%, *p* = 0.0182, Figure 3(g)) in the HFD group compared to the control group was noticed. On the other hand, in HFD rats, a notably higher saliva/serum ratio of 4-HNE (↑66%, *p* = 0.0008, Figure 3(o)) was observed compared to the control group.

Interestingly, significant positive correlations of all of the oxidative and nitrosative damage products such as PC, 3-NT, AGE, and 4-HNE (*r* = 0.4727, *p* = 0.0353, Figure 3(d); *r* = 0.4789, *p* = 0.0327, Figure 3(h); *r* = 0.4777, *p* = 0.0331, Figure 3(l); *r* = 0.8341, *p* ≤ 0.0001, Figure 3(p), respectively) were observed between plasma and saliva in the HFD group.

### 2.5. Correlations with Metabolic Parameters

**Figure 4:**
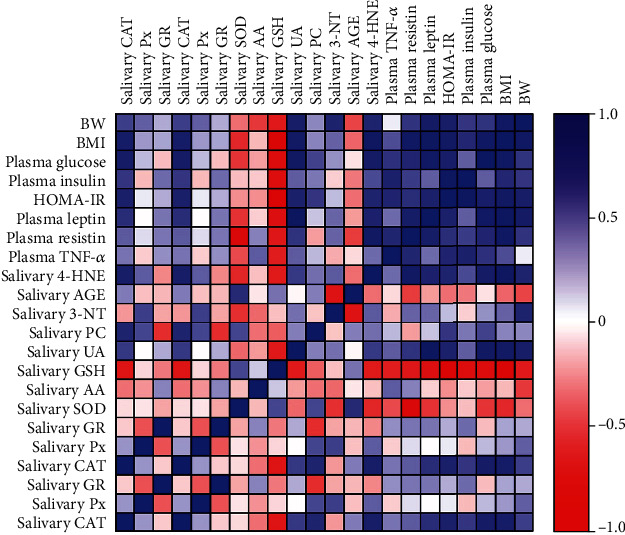
Correlations between salivary and plasma redox and metabolic parameters in rats. Abbreviations: 3-NT: 3-nitrotyrosine; 4-HNE: 4-hydroxynonenal; AA: ascorbic acid; AGE: advanced glycation end products; BMI: body mass index; BW: body weight; CAT: catalase; GSH: reduced glutathione; GR: glutathione reductase; HOMA-IR: homeostatic model assessment for insulin resistance; PC: protein carbonyls; Px: peroxidase; SOD: superoxide dismutase; TNF-*α*: tumor necrosis factor *α*; UA: uric acid.

The analysis of the metabolic parameters and investigated biomarkers revealed highly positive correlations between salivary CAT and salivary 4-HNE, as well as plasma glucose HOMA-IR, and BMI (*r* = 0.693, *p* = 0.026; *r* = 0.651, *p* = 0.041; *r* = 0.712, *p* = 0.021; *r* = 0.715, *p* = 0.02, respectively). Interestingly, the activity of salivary SOD was correlated only with plasma resistin (*r* = −0.851, *p* = 0.002). Moreover, the activity of salivary GSH correlated negatively with BW (*r* = −0.667, *p* = 0.035) and BMI (*r* = −0.889, *p* = 0.001), as well as plasma glucose (*r* = −0.79, *p* = 0.006), insulin (*r* = −0.783, *p* = 0.007), HOMA-IR (*r* = −0.936, *p* = 0.00007), and proinflammatory adipokines (leptin: *r* = −0.757, *p* = 0.011; resistin: *r* = 0.685, *p* = 0.029; TNF-*α*: *r* = −0.639, *p* = 0.047). What is more, we have shown highly positive correlation between the salivary UA concentration and plasma leptin, plasma glucose, BW, and BMI (*r* = 0.92, *p* ≤ 0.0001; *r* = 0.777, *p* = 0.008; *r* = 0.691, *p* = 0.027; *r* = 0.754, *p* = 0.012, respectively). Furthermore, we observed the positive correlations of salivary 4-HNE and resistin (*r* = 0.776, *p* = 0.008). However, salivary 4-HNE correlated also with BW (*r* = 0.605, *p* = 0.044) and BMI (*r* = 0.854, *p* = 0.002), as well as indicators of carbohydrate metabolism-glucose (*r* = 0.716, *p* = 0.02) and HOMA-IR (*r* = 0.592, *p* = 0.05) (Figure 4).

### 2.6. Multiple Regression Analysis

Multiple regression analysis of salivary biomarkers showed that salivary GSH was negatively associated with HOMA-IR (*p* = 0.0042). Moreover, significant association between BMI and the activity of salivary GSH and concentration of salivary 4-HNE were observed. No significant associations between other salivary biomarkers and BMI or HOMA-IR were noticed (Table 2).

### 2.7. ROC Analysis of the Analyzed Redox Biomarkers

The ROC analysis revealed that most of the salivary redox biomarkers significantly differentiated rats fed a standard diet and a high-fat diet. The assessment of salivary Px, SOD, and GSH (sensitivity = 70%, specificity = 70%, *p* = 0.0102; sensitivity = 90%, specificity = 90%, *p* = 0.0004; sensitivity = 100%, specificity = 100%, *p* = 0.0002) clearly distinguished the HFD group from the control. Similarly, the salivary levels of AA, UA, PC, 3-NT, AGE, and 4-HNE (sensitivity = 90%, specificity = 90%, *p* = 0.0002; sensitivity = 80%, specificity = 80%, *p* = 0.0025; sensitivity = 80%, specificity = 80%, *p* = 0.0032; sensitivity = 70%, specificity = 70%, *p* = 0.0343; sensitivity = 90%, specificity = 90%, *p* = 0.0009; sensitivity = 100%, specificity = 100%, *p* = 0.0002) significantly differentiated the HFD rats from the normal diet group. Moreover, number of serum and plasma biomarkers, such as CAT, SOD, GSH, and 4-HNE (*p* = 0.0002), were characterized by a 100% of sensitivity and specificity in differentiating the HFD rats from the control rats (Table 3).

## 3. Discussion

This study is the first to evaluate the clinical utility of salivary redox biomarkers in IR. In preclinical studies in an animal model, we demonstrated that salivary GSH and 4-HNE levels have high diagnostic value in monitoring IR progression.

IR is a major pathogenetic factor in DM2 preceding the onset of overt hyperglycemia by up to several years [3]. Although decreased tissue sensitivity to insulin is compensated by hyperinsulinemia, IR leads to obesity, hypertension, dyslipidemia, and finally the metabolic syndrome [4]. Therefore, understanding the causes of IR and its treatment is one of the most significant challenges in modern diabetology. The gold standard for IR assessment is the hyperinsulinemic-euglycemic clamp technique [31]. However, this method is not used in routine medical practice due to its high invasiveness and the need to perform the test while the patient is hospitalized. For this reason, insulin resistance is mainly diagnosed using indirect methods. The simplest of them is a measurement of fasting glucose and insulin and calculation of HOMA-IR index (homeostatic model assessment of IR), which is a mathematical model describing interdependence of insulin secretion in response to current basal glycemia [3, 31]. Nevertheless, new biomarkers are still being sought that could noninvasively inform about the progression of IR and its metabolic complications [1]. Of particular diagnostic interest is saliva, which is easy to collect and does not require the specialized equipment or assistance of the medical staff. Saliva can be collected multiple times per day and can replace blood draws in people with clotting disorders, children, or patients with disabilities [24, 25]. Noninvasive saliva collection reduces patient anxiety, promotes more frequent self-monitoring, and enables the disease diagnosis at an early stage [32]. Unfortunately, the assessment of glucose and insulin in the saliva is not diagnostically relevant because it does not reflect their levels in the blood [33–35]. Nonetheless, there has been considerable recent interest in saliva-based diagnostics focusing on redox biomarkers. Salivary redox biomarkers are commonly used to diagnose hypertension [36, 37], obesity [38–40], chronic kidney disease [41, 42], heart failure [43, 44], Hashimoto's disease [45, 46], dementia [47, 48], or cancer [49, 50]. Considering the critical contribution of oxidative stress in the progression of IR [6, 9, 10, 15, 16], we evaluated the salivary antioxidants and products of protein and lipid oxidation/nitration in the saliva of IR rats. We compared the content of salivary redox biomarkers with their plasma levels and the classical indicators of metabolic disorders. We also assessed the diagnostic utility of salivary redox biomarkers using ROC analysis and multivariate regression.

We have shown that IR results in impairment of the salivary/plasma antioxidant barrier, with both enzymatic (↓SOD, ↓SPx/GPx) and nonenzymatic (↓GSH, ↓AA, ↑UA) deficiency. Although we did not assess the rate of ROS production, the weakening of antioxidant systems is likely due to increased free radical generation under IR conditions [44]. It is well known that positive energy balance (↑fat supply) causes increased synthesis of acetyl-CoA and NADP in mitochondria, responsible for ROS overproduction [51, 52]. However, adipose tissue is also an essential source of free radicals in IR [53]. Excess visceral tissue stimulates adipocytes to synthesize chemotactic and adhesive molecules such as MCP1 (monocyte chemoattractant protein 1), VCAM1 (vascular cell adhesion molecule 1), and ICAM (intercellular adhesion molecule 1), which enhance the influx of lymphocytes and macrophages and stimulate the production of proinflammatory cytokines (IL-1, IL-2, TNF-*α*) [53–55]. Synthesis of adipokines (resistin and leptin), which mediate inflammation by promoting cytokine efflux, is also increased [53]. Thus, it is not surprising that the antioxidant barrier is diminished, resulting in higher oxidative damage to proteins (↑PC, ↑3-NT, ↑AGE) and lipids (↑4-HNE). Of particular note are disturbances in glutathione metabolism (↓GSH, ↓SPx/GPx). GSH participates in hydrogen peroxide degradation and maintains the sulfhydryl groups of proteins in a reduced state [56]. The accumulation of oxidized glutathione (GSSG) in the cell and the formation of protein disulfides with GSH inhibits many enzymes' activity, thereby impairs cell metabolism and energy production [56, 57]. The increased oxidation (↑PC, ↑4-HNE), glycation (↑AGE), and nitration (↑3-NT) of proteins/lipids observed in our study are also very important. The products of oxidative/nitrosative modifications damage cellular structures and show mutagenic and carcinogenic properties. Indeed, compounds such as 4-HNE can form adducts with DNA, promoting instability of the genetic material and several replication errors [58, 59].

A crucial part of our study was to evaluate the diagnostic utility of salivary redox biomarkers in IR conditions. Biomarkers are biological indicators whose assessment allows qualitative or quantitative evaluation of pathological states and diseases. The biomarkers should differentiate patients from healthy controls (with high accuracy and specificity) and correlate with disease severity [23]. Of all the biomarkers we evaluated, salivary GSH and 4-HNE deserve special attention. Salivary GSH correlates negatively not only with body weight (*r* = −0.667, *p* = 0.035) and BMI (*r* = −0.889, *p* = 0.001), but also with plasma glucose (*r* = −0.79, *p* = 0.006), insulin (*r* = −0.783, *p* = 0.007), HOMA-IR (*r* = −0.936, *p* = 0.00007), and proinflammatory adipokines (leptin: *r* = −0.757, *p* = 0.011; resistin: *r* = 0.685, *p* = 0.029; and TNF-*α*: *r* = −0.639, *p* = 0.047). Multivariate regression analysis also showed that salivary GSH depends on the severity of obesity measured by BMI and reduced insulin sensitivity expressed as HOMA-IR. Therefore, GSH depletion may be associated with the progression of metabolic disturbances accompanying insulin resistance. Indeed, glutathione is one of the most critical intracellular antioxidants [22, 60]. In addition to ROS scavenging and regenerating other antioxidants (e.g., vitamin E and GPx), GSH participates in restoring oxidatively modified proteins, lipids, and nucleic acids. It also acts as a major thiol buffer of the cell by regulating growth, differentiation, and apoptosis [56, 57]. Reduced GSH level is a critical factor in increasing the intensity of membrane lipid peroxidation and ceramide accumulation in patients with obesity and IR [22, 61, 62]. In our study, this may be supported by the negative correlation between salivary GSH and 4-HNE (*r* = 0.663, *p* = 0.037). Indeed, lipids are particularly susceptible to oxidation. Lipid peroxidation products such as 4-HNE cause further cellular damage, including disruption of gene expression/protein synthesis and uncoupling of oxidative phosphorylation [63]. 4-HNE can also increase inflammation by stimulating NADPH oxidase activity or activating macrophages [58, 64]. Therefore, the positive correlations of salivary 4-HNE and adipokines are not surprising (leptin: *r* = 0.626, *p* = 0.053; and resistin: *r* = 0.776, *p* = 0.008). However, salivary 4-HNE correlates also with body weight (*r* = 0.605, *p* = 0.044) and BMI (*r* = 0.854, *p* = 0.002), as well as indicators of carbohydrate metabolism (glucose (*r* = 0.716, *p* = 0.02); and HOMA-IR (*r* = 0.592, *p* = 0.05)). It is well known that IR is the most important cause of carbohydrate disorders. Hyperinsulinemia and insulin resistance are also independent factors in diabetes and cardiovascular disease [4, 5]. Therefore, we used multiple regression to determine the diagnostic utility of salivary 4-HNE in the HFD-induced IR model. Regression analysis showed that this parameter highly depends on the HOMA-IR index and BMI. Its positive correlation with proinflammatory adipokines also demonstrates the diagnostic utility of salivary 4-HNE.

The oral cavity is a unique site in the body since it is exposed to many prooxidant factors such as air pollutants, diet, medications, dental materials, and other xenobiotics [65, 66]. Although many antioxidants/oxidative stress products pass into saliva from the blood, their salivary content can not necessarily reflect the intensity of systemic oxidative stress. Indeed, in our study, salivary antioxidant enzymes did not correlate with serum activity (exception: SOD). Nevertheless, salivary nonenzymatic antioxidants reflect very well their plasma levels. We also observed positive correlations between the concentrations of protein and lipid oxidation products in plasma and saliva of IR rats. However, the highest correlation coefficients are found for GSH (*r* = 0.7993, *p* < 0.0001) and 4-HNE (*r* = 0.8341, *p* < 0.0001). Therefore, salivary GSH and 4-HNE can be used to assess systemic redox homeostasis. The results of the ROC analysis also demonstrated the high diagnostic utility of these biomarkers. ROC analysis evaluates the diagnostic power of the test and assesses the ability of the biomarker to discriminate between normal and abnormal values. Salivary GSH and 4-HNE differentiate with very high sensitivity (100%) and specificity (100%) between healthy animals and those with IR (AUC = 1.0). Thus, salivary GSH and 4-HNE meet all the criteria for a good laboratory biomarker [23]. Further clinical studies are needed to evaluate their diagnostic potential. However, it should not be forgotten that in IR there is salivary hypofunction caused by disturbances in redox homeostasis [12, 67, 68], which may affect the oxidative stress parameters in saliva. It is also necessary to compare salivary redox biomarkers in patients with IR and those with other metabolic, cardiovascular, and inflammatory diseases [44].

It is essential to note the limitations of our work. Because of the lack of Ethics Committee approval, we could not perform a hyperinsulinemic-euglycemic clamp. Additionally, we evaluated only the most commonly assessed redox biomarkers due to the low volume of saliva. Although the redox biomarkers described here distinguish IR rats and controls with high specificity and sensitivity, they may be nonspecific only for insulin resistance. Therefore, it is essential to evaluate salivary redox indicators also in other diseases with oxidative stress etiology. Nevertheless, this is the first study to show the potential use of saliva and oxidative stress biomarkers in monitoring IR progression.

## 4. Conclusions

1. Saliva can be used to assess the systemic antioxidant status and the intensity of systemic oxidative stress
2. Salivary GSH and 4-HNE may be potential biomarkers of IR progression
3. There is a need for human clinical trials to evaluate the diagnostic utility of salivary redox biomarkers in IR conditions

## 5. Materials and Methods

### 5.1. Animals

The experiment was performed on male Wistar rats (*R. norvegicus*; Wistar: cmd, outbred Cmdb:Wi) with an initial body weight of 50–60 g. The animals came from the Center for Experimental Medicine of the Medical University of Bialystok. The rats have been housed in individually ventilated laboratory cages at controlled temperatures (20–22°C), under a standard condition of light from 6.00 a.m. to 6.00 p.m., and with free access to tap water and food.

The experimental procedures were approved by the institutional Committee for Ethics use of Animals in the University of Warmia and Mazury in Olsztyn, Poland (No. 21/2017).

After seven days of adaptation, the rats were divided into two groups of 10 individuals each.

(i) Group I—(C) control; rats receiving standard rodent diet (Research Diets, New Brunswick, NJ, USA, catalog number D12450J) containing 10% fat, 20% proteins, and 70% carbohydrates

(ii) Group II—(HFD) rats fed a high-fat diet (Research Diets, New Brunswick, NJ, USA catalog number D12492) containing 60% fat, 20% proteins, and 20% carbohydrates

Animals from control and HFD groups were fed the appropriate diet for eight weeks, while the body weight and food intake were monitored weekly. The body mass index (BMI) was calculated using the formula BMI = body weight (g)/length^2^ (cm^2^), and rat length was measured from the tip of the nose to the anus. BMI between 0.45 and 0.68 g/cm^2^ was considered normal values, whereas BMI greater than 0.68 g/cm^2^ indicated obesity [69, 70].

After eight weeks of the experiment, rats were fasted for 12 h, anesthetized with sodium phenobarbital (80 mg/kg body weight, intraperitoneally), and then the whole saliva was collected. The animals were peritoneally injected with 5 mg/kg BW pilocarpine nitrate (Sigma Chemical Co; St. Louis, MO, USA) in physiological saline. Five minutes later, a preweighted cotton ball was inserted into the oral cavity, and saliva was collected for five minutes [67, 68]. The volume of saliva was evaluated by subtracting the initial weight of cotton balls from their final weight. One mg of the collected saliva was considered to be one *μ*L [67, 68]. Saliva was then centrifuged in Salivette tubes (3000 × g, 4°C, 10 min) to collect supernatant [71]. Next, whole blood was collected from the abdominal aorta into glass tubes (to obtain serum) and EDTA tubes (to obtain plasma) and centrifuged (3000 × g, 4°C, 10 min). To protect against sample oxidation and proteolysis, the antioxidant butylated hydroxytoluene (BHT, ten *μ*L of 0.5 M BHT in acetonitrile per 1 mL sample; Sigma-Aldrich, Steinheim, Germany) and a protease inhibitor (Complete Mini Roche, France) were added to the collected saliva and plasma samples [72, 73]. All samples were stored at -80°C but for no longer than six months.

Fasting blood glucose level was determined using the glucometer (Accu-Chek; Bayer, Germany). Fasting plasma insulin level was determined using a commercial ELISA kit according to the manufacturer's instructions (EIAab Science Inc. Wuhan; Wuhan, China). To confirm IR, the insulin sensitivity was determined using the homeostasis model assessment (HOMA − IR) = fasting insulin (U/mL) × fasting glucose (mM)/22.5 [74]. According to the manufacturer's instructions, plasma adipocytokines (leptin, resistin, and TNF-*α*) were determined using a commercial ELISA kit (EIAab Science Inc. Wuhan; Wuhan, China).

### 5.2. Redox Assays

#### 5.2.1. Enzymatic Antioxidants

The activity of catalase (CAT) was analyzed spectrophotometrically by measuring the decomposition rate of hydrogen peroxide (H_2_O_2_) in the sample at 240 nm wavelength [75]. One CAT unit was expressed as the amount of enzyme that decomposes 1 mmol H_2_O_2_ within 1 minute. Salivary peroxidase (Px) activity was determined spectrophotometrically according to Mansson-Rahemtulla et al. method [76]. The absorbance changes in the reaction mixture containing 5,5′-dithiobis-2-nitrobenzoic acid (DTNB), potassium iodide (KI), and H_2_O_2_ were measured at 412 nm wavelength. The activity of glutathione peroxidase (GPx) was assayed spectrophotometrically by the Paglia and Valentine method [77]. The absorbance was analyzed at 340 nm, based on the conversion of reduced nicotinamide adenine dinucleotide phosphate (NADPH) to reduced nicotinamide adenine dinucleotide phosphate (NADP+). One unit of GPx activity was assumed to catalyze the oxidation of 1 mmol of NADPH for 1 minute. The method of Mize and Langdon [78] was used to assess glutathione reductase (GR) activity. The absorbance of the samples was measured at 340 nm wavelength. One unit of GR activity was expressed as the amount of enzyme needed for the oxidation reaction of 1 *μ*mol of NADPH within 1 minute. The activity of superoxide dismutase (SOD) was determined spectrophotometrically by measuring the absorbance changes accompanying adrenaline oxidation at 480 nm wavelength [79]. One unit of SOD activity was assumed to inhibit the oxidation of adrenaline by 50%.

#### 5.2.2. Nonenzymatic Antioxidants

The concentration of ascorbic acid (AA) was analyzed colorimetrically according to Jagota and Dani [80]. This method involves the reduction of the Folin phenol reagent under the influence of AA. The absorbance of the samples was measured at 760 nm wavelength. The content of reduced glutathione (GSH) was measured spectrophotometrically based on the reduction of DTNB to 2-nitro-5-mercaptobenzoic acid under the influence of GSH. The absorbance was measured at 412 nm wavelength [81]. The concentration of uric acid (UA) was analyzed colorimetrically by measuring the absorbance of 2,4,6-tripyridyl-s-triazine complex with iron ions and UA, using the commercial kit QuantiChromTM Uric Acid DIUA-250 (BioAssay Systems, Harward, CA, USA). The intensity of the examined sample was measured at 490 nm wavelength.

#### 5.2.3. Oxidative/Nitrosative Damage

The concentration of protein carbonyl (PC) was determined colorimetrically based on the 2,4-dinitrophenylhydrazine (2,4-DNPH)'s reaction with carbonyl groups in the oxidatively damaged proteins. The intensity of the resultant hydrazone was measured at 355 nm. PC content was calculated using an absorption coefficient for 2, 4 − DNPH = 22,000 M^−1^ cm^−1^. According to the manufacturer's instructions, the concentration of 3-nitrotyrosine (3-NT) was measured by the ELISA method, using a commercial diagnostic kit (Immundiagnostik AG; Bensheim, Germany). Advanced glycation end product (AGE) level was analyzed spectrofluorimetrically by measuring the specific AGE fluorescence at 350/440 nm [82]. The samples were diluted in 0.02 M PBS buffer for the AGE determination in plasma [44]. The concentration of 4-hydroxynonenal protein adduct (4-HNE) was determined by the ELISA method (OxiSelect™HNE Adduct Competitive ELISA Kit, Cell Biolabs Inc. San Diego, CA, USA), following the manufacturer's instructions provided in the package.

### 5.3. Statistical Analysis

Statistical analysis was performed using GraphPad Prism 8.4.3 for MacOS (GraphPad Software, La Jolla, USA). The Shapiro–Wilk test was used to determine the normality of distribution, while the Student's *t*-test was used to compare the IR group with the controls. The results were presented as mean ± standard deviation (SD), and the value of *p* < 0.05 was considered statistically significant. Pearson correlation coefficient was used to evaluate the relationships between redox biomarkers and metabolic parameters. To identify factors that determine the levels of redox biomarkers, we performed multiple regression analyses. HOMA-IR and BMI were included as independent variables; 95% confidence intervals (CI) were reported along with regression parameters. Receiver operating characteristic (ROC) analysis was used to assess the diagnostic utility of the redox biomarkers. AUC (area under the curve) and optimal cut-off values were determined for each parameter that ensured high sensitivity with high specificity.

The number of animals was calculated a priori based on our previous preliminary study. Type I error *α* = 0.05 and statistical power (type II error) of 0.9 were considered. Statistical test assumptions were validated for all the analyses performed. The minimum number of rats in one group was seven, and therefore, the analysis was performed on ten individuals.

## Figures and Tables

**Table 1:** General characteristics of the control and high-fat diet fed (HFD) rats.

Parameter	Control group	HFD group
Body weight (g)	388 ± 34.16	537 ± 36.52^∗∗∗∗^
BMI (g/cm^2^)	0.66 ± 0.024	0.90 ± 0.016^∗∗∗∗^
Energy intake (g/rat/week)	206.4 ± 22.06	248.6 ± 38.23^∗∗^
Food intake (g/day)	18.07 ± 1.93	11.05 ± 1.70^∗∗∗∗^
Plasma glucose (mg/dL)	90.94 ± 3.31	146.4 ± 12.54^∗∗∗∗^
Plasma insulin (mU/mL)	78.65 ± 7.55	172.30 ± 12.07^∗∗∗∗^
HOMA-IR	1.72 ± 0.18	16.20 ± 0.65^∗∗∗∗^
Plasma leptin (mU/mL)	26.84 ± 2.88	49.40 ± 6.24^∗∗∗∗^
Plasma resistin (mU/mL)	184 ± 19.12	394 ± 39.51^∗∗∗∗^
Plasma TNF-*α* (mU/mL)	1010 ± 115.2	3248 ± 588.5^∗∗∗∗^

Abbreviations: BMI: body mass index; HFD: high-fat diet; HOMA-IR: homeostatic model assessment of insulin resistance; TNF-*α*: tumor necrosis factor *α*; ^∗∗^*p* < 0.01 vs. control; ^∗∗∗∗^*p* < 0.0001 vs. control.

**Table 2:** Multiple regression analysis of the analyzed redox biomarkers.

Parameter	*β*1: HOMA-IR			*β*2: BMI		
	Estimate	95% CI	*p* value	Estimate	95% CI	*p* value
Salivary CAT	0.03866	-0.1186 to 0.1959	0.5793	0.7877	-0.6970 to 2.272	0.2499
Salivary Px	-4.2	-23.23 to 14.83	0.6178	57.27	-122.3 to 236.9	0.4754
Salivary GR	0.07613	-1.587 to 1.740	0.9169	1.356	-14.35 to 17.06	0.844
Salivary SOD	0.02902	-0.02624 to 0.08427	0.2544	-0.4925	-1.014 to 0.02913	0.0607
Salivary AA	-1.039	-5.541 to 3.462	0.602	3.56	-38.93 to 46.05	0.8486
Salivary GSH	-0.2338	-0.3665 to -0.1011	0.0042	-1.44	-2.693 to -0.1877	0.0298
Salivary UA	0.01866	-0.1543 to 0.1916	0.806	1.175	-0.4573 to 2.808	0.1325
Salivary PC	0.2107	-0.1228 to 0.5443	0.1788	-0.9228	-4.071 to 2.226	0.5106
Salivary 3-NT	-0.2589	-1.162 to 0.6447	0.5198	4.142	-4.387 to 12.67	0.2885
Salivary AGE	-0.01719	-0.2152 to 0.1808	0.8432	-0.3393	-2.209 to 1.530	0.6806
Salivary 4-HNE	-0.9857	-4.660 to 2.688	0.546	47.96	13.29 to 82.64	0.0137

Abbreviations: 3-NT: 3-nitrotyrosine; 4-HNE: 4-hydroxynonenal; AA: ascorbic acid; AGE: advanced glycation end products; BMI: body mass index; CAT: catalase; CI: confidence interval; GSH: reduced glutathione; GR: glutathione reductase; HOMA-IR: homeostatic model assessment for insulin resistance; PC: protein carbonyls; Px: peroxidase; SOD: superoxide dismutase; UA: uric acid.

**Table 3:** ROC analysis of the analyzed redox biomarkers.

Biomarker	Saliva								Serum/plasma							
	AUC	95% CI	*p* value	Cut-off	Sensitivity %	95% CI	Specificity %	95% CI	AUC	95% CI	*p* value	Cut-off	Sensitivity %	95% CI	Specificity %	95% CI
CAT	0.76	0.5360 to 0.9840	0.0494	>0.5592	60	31.27% to 83.18%	60	31.27% to 83.18%	1	1.000 to 1.000	0.0002	<1.244	100	72.25% to 100.0%	100	72.25% to 100.0%
Px/GPx	0.84	0.6581 to 1.000	0.0102	<29.80	70	39.68% to 89.22%	70	39.68% to 89.22%	0.63	0.3657 to 0.8943	0.3258	<35.10	50	23.66% to 76.34%	50	23.66% to 76.34%
GR	0.57	0.3009 to 0.8391	0.5967	<1.719	50	23.66% to 76.34%	50	23.66% to 76.34%	0.6	0.3419 to 0.8581	0.4497	<10.62	60	31.27% to 83.18%	60	31.27% to 83.18%
SOD	0.97	0.9071 to 1.000	0.0004	<0.2066	90	59.58% to 99.49%	90	59.58% to 99.49%	1	1.000 to 1.000	0.0002	<0.5534	100	72.25% to 100.0%	100	72.25% to 100.0%
AA	0.99	0.9583 to 1.000	0.0002	<9.841	90	59.58% to 99.49%	90	59.58% to 99.49%	0.89	0.7500 to 1.000	0.0032	<8.703	80	49.02% to 96.45%	80	49.02% to 96.45%
GSH	1	1.000 to 1.000	0.0002	<0.9395	100	72.25% to 100.0%	100	72.25% to 100.0%	1	1.000 to 1.000	0.0002	<0.4547	100	72.25% to 100.0%	100	72.25% to 100.0%
UA	0.9	0.7609 to 1.000	0.0025	>0.4447	80	49.02% to 96.45%	80	49.02% to 96.45%	0.86	0.6739 to 1.000	0.0065	>0.3867	80	49.02% to 96.45%	80	49.02% to 96.45%
PC	0.89	0.7476 to 1.000	0.0032	>0.3696	80	49.02% to 96.45%	80	49.02% to 96.45%	0.95	0.8477 to 1.000	0.0007	>0.4784	90	59.58% to 99.49%	90	59.58% to 99.49%
3-NT	0.78	0.5536 to 1.000	0.0343	>6.415	70	39.68% to 89.22%	70	39.68% to 89.22%	0.91	0.7557 to 1.000	0.0019	>11.01	90	59.58% to 99.49%	90	59.58% to 99.49%
AGE	0.94	0.8208 to 1.000	0.0009	>0.4471	90	59.58% to 99.49%	90	59.58% to 99.49%	0.95	0.8622 to 1.000	0.0007	>1.192	80	49.02% to 96.45%	80	49.02% to 96.45%
4-HNE	1	1.000 to 1.000	0.0002	>8.293	100	72.25% to 100.0%	100	72.25% to 100.0%	1	1.000 to 1.000	0.0002	>54.87	100	72.25% to 100.0%	100	72.25% to 100.0%

Abbreviations: 3-NT: 3-nitrotyrosine; 4-HNE: 4-hydroxynonenal; AA: ascorbic acid; AGE: advanced glycation end products; AUC: area under the curve; CAT: catalase; CI: confidence interval; GPx: glutathione peroxidase; GSH: reduced glutathione; GR: glutathione reductase; HOMA-IR: homeostatic model assessment for insulin resistance; PC: protein carbonyls; Px: peroxidase; ROC: receiver operating characteristic; SOD: superoxide dismutase; UA: uric acid.

## Data Availability

The datasets generated for this study are available on request to the corresponding author.
